# Sorafenib inhibits LPS-induced inflammation by regulating Lyn-MAPK-NF-kB/AP-1 pathway and TLR4 expression

**DOI:** 10.1038/s41420-022-01073-7

**Published:** 2022-06-09

**Authors:** Xiaolian Li, Mingkun Xu, Jiaojiao Shen, Yuqin Li, Shaoping Lin, Min Zhu, Qiongni Pang, Xiujuan Tan, Jing Tang

**Affiliations:** 1 Department of Anesthesiology, Affiliated Hospital of Guangdong Medical University, 524000 Zhanjiang City, Guangdong Province China; 2 Department of Anesthesiology, Guangdong Provincial People’s Hospital, Guangdong Academy of Medical Sciences, 510080 Guangzhou City, Guangdong Province China

**Keywords:** Sepsis, Viral infection

## Abstract

Sorafenib is an anti-tumor drug widely used in clinical treatment, which can inhibit tyrosine kinase receptor on cell surface and serine/threonine kinase in downstream Ras/MAPK cascade signaling pathway of cells. Tyrosine kinase phosphorylation plays an important role in inflammatory mechanism, such as TLR4 tyrosine phosphorylation, MAPK pathway protein activation, and activation of downstream NF-кB. However, the effects of sorafenib on LPS-induced inflammatory reaction and its specific mechanism have still remained unknown. We found that sorafenib inhibited the phosphorylation of tyrosine kinase Lyn induced by LPS, thereby reducing the phosphorylation level of p38 and JNK, inhibiting the activation of c-Jun and NF-κB, and then inhibiting the expression of inflammatory factors IL-6, IL-1β, and TNF-α. Furthermore, sorafenib also decreased the expression of TLR4 on the macrophage membrane to inhibit the expression of inflammatory factors latterly, which may be related to the inactivation of Lyn. These results provide a new perspective and direction for the clinical treatment of sepsis.

## Introduction

Sepsis, a syndrome of physiologic, pathologic, and biochemical abnormalities induced by infection, refers to the life-threatening organ dysfunction, which is a leading cause of mortality and critical illness worldwide [1–3]. It is estimated that more than 30 million people are hospitalized for sepsis every year in the world, and sepsis may cause as many as 5.3 million deaths every year [3]. Although sepsis is associated with high morbidity and mortality, there are few effective therapies to date [4, 5]. At present, antibiotics and symptomatic treatment of complications are the main clinical treatments for sepsis. Antibiotics can effectively kill bacteria, but the internal and external toxins, bacterial DNA and cell wall components released by dead bacteria could cause an excessive inflammatory reaction, and then lead to multiple organ failure and death [6]. Therefore, in the treatment of sepsis, it is particularly important to inhibit inflammation on the basis of antibiosis.

The recognition of bacterial lipopolysaccharide (LPS), RNA, or DNA by TLR receptor activates the pro-inflammatory cytokine profile in macrophages, thus destroying the dynamic balance regulation of the immune system [7]. Src family kinase (SFKs) is a non-receptor protein tyrosine kinase family composed of nine members: Src, Lyn, Fgr, Hck, Lck, Fyn, Blk, Yes, and Ylk. As a protein tyrosine kinase, SFK is involved in the regulation of many cell functions, such as proliferation, migration, differentiation, survival, immune response of B lymphocytes and T lymphocytes, and perception of microorganisms by pattern-recognition receptors [8, 9]. Lyn is the most abundant Src family kinase in B cells, which is closely related to the B-cell receptor complex. Besides, Lyn also exists in many hematopoietic cells [10–12]. What’s more, Lyn is a key regulator of the mucosal immune system, which plays an important role in mucus secretion of airway inflammation and airway remodeling, and can reduce excessive secretion of airway mucus and strictly regulate airway remodeling [13, 14]. In the case of Lyn knockout, the activation of the MAPK pathway was impaired and the activation of IKK-NF-κB pathway triggered by TLR4 was also inhibited after LPS stimulation [15–17].

Tyrosine kinase phosphorylation plays an important role in inflammatory mechanism, such as TLR4 tyrosine phosphorylation, activation of MAPK pathway protein and downstream NF-кB [18–20]. Sorafenib (BAY 43-9006) is a multikinase inhibitor with anti-tumor activity in various tumor models [21], which can inhibit the Raf/MEK/ERK signaling pathway [22], and inhibit receptor tyrosine kinases which promote angiogenesis, such as vascular endothelial growth factor receptor (VEGFR)1, VEGFR2, VEGFR 3, platelet-derived growth factor receptor -β(PDGFR-β), Flt3 and c-Kit, playing a key role in tumor cell proliferation and tumor angiogenesis [23–25]. Studies have shown that sorafenib can not only inhibit the proliferation and angiogenesis of tumor cells but also regulate the function of immune cells. In addition to inhibiting the phenotype and function of dendritic cells, sorafenib also inhibits caspase-1 overexpression through limiting nuclear transport of p65, which leads to the inactivation of NF-κB [26, 27]. However, the effect of sorafenib, a tyrosine kinase inhibitor, on LPS-induced inflammatory reaction and its specific mechanism are still remained unknown.

## Results

### Sorafenib inhibits pro-inflammatory cytokines induced by LPS in macrophages

**Fig. 1:**
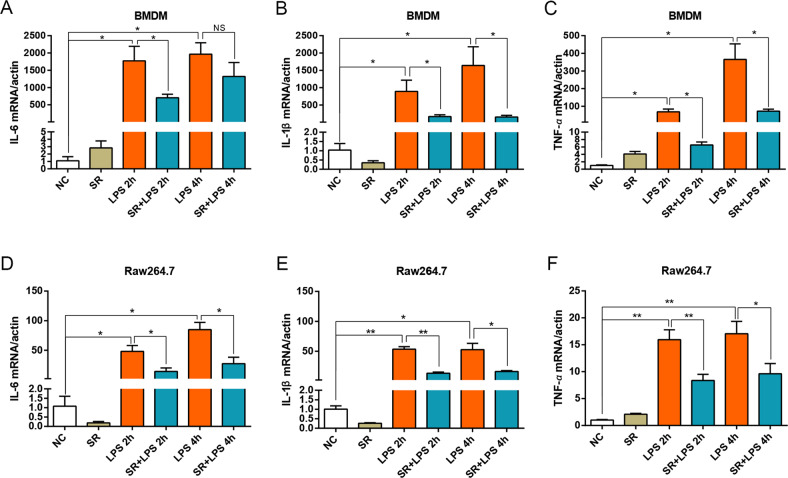
Pretreatment of sorafenib inhibits LPS-induced inflammatory response in macrophage. mRNA expression levels of IL-6, IL-1β, and TNF-α were measured by quantitative PCR in BMDMs which pretreated with 10 μmol/L sorafenib for 30 min prior to LPS (1 μg/mL) treatment for 2 and 4 h (**A**–**C**). The same method was verified on Raw264.7 **D**–**F**. All mRNA levels were normalized to the level of β-actin mRNA. The data are expressed as the means ± SEM of three independent experiments. ns, *P* > 0.05; *P < 0.05; **P < 0.01 compared with other groups.

We first determined the effects of sorafenib on LPS-induced inflammatory response. BMDM and RAW264.7 cells were pretreated with 10 μmol/L sorafenib for 30 min, and then 1 μg/mL LPS was added to stimulate the inflammatory response of macrophages for 2 or 4 h, respectively. The mRNA expression levels of pro-inflammatory cytokines including IL-6, IL-1β, and TNF-α were determined by real-time qPCR. As shown in Fig. 1A–C, the mRNA expression of IL-6, IL-1β, and TNF-α were significantly upregulated in LPS-treated BMDMs in a time-dependent manner compared with the control group, while the mRNA expression of IL-6, IL-1β, and TNF-α were weakened as expected after the pretreatment of sofafenib. The same results were observed in RAW264.7 cells (Fig. 1D–F). The aforementioned evidence suggested that sorafenib has inhibitory effects on LPS-induced inflammatory reaction in macrophages.

### Sorafenib alleviates LPS-induced inflammation in vivo

**Fig. 2:**
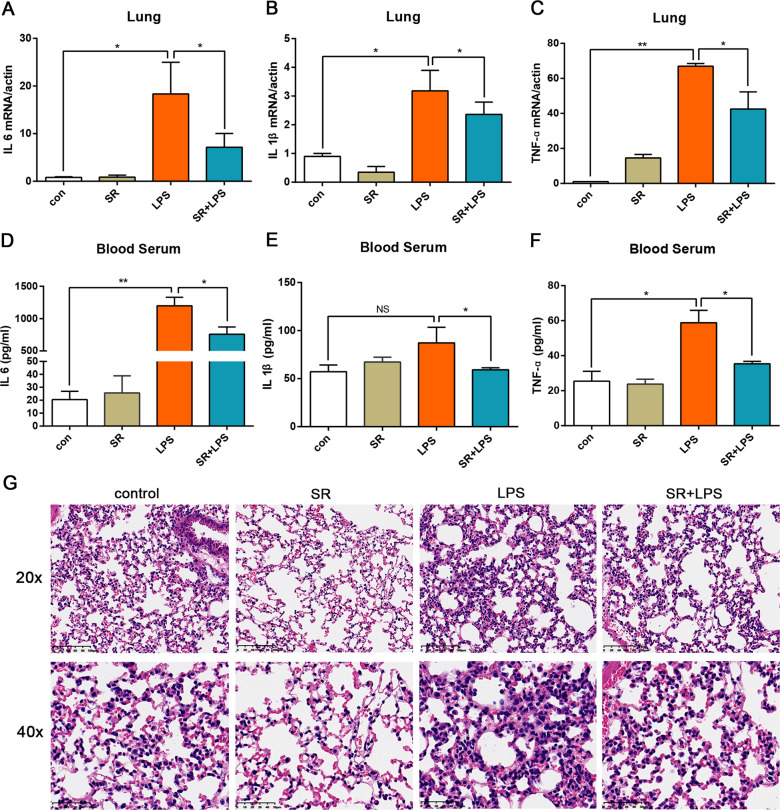
Sorafenib alleviates LPS-induced inflammation in vivo. In all, 6–8 W WT mice were + /− pretreated with 100 mg/kg sorafenib by intragastric administration for 2 h, and then LPS (20 mg/kg) were injected intraperitoneally for 12 h to establish the inflammatory model. The transcription levels of IL-6 **A**, IL-1β (**B**), and TNF-α (**C**) in lung tissue were detected by qRT-PCR and protein levels in serum were measured by ELISA **D**–**F**. H&E staining microscopy images (magnification ×20 and 40) of lung sections of mice in the control group and different treatment groups were shown in (**G**). All images are representative of at least three independent experiments. The graphs show means ± SEM, *n* = 3; *P < 0.05; **P < 0.01 compared with other groups.

To establish an inflammatory mouse model, aged 6–8 weeks C57BL/6 mice were injected with 20 mg/kg LPS intraperitoneally. The mice injected with saline were used as controls. To explore the effect of sorafenib on LPS-induced inflammation in mice, 100 mg/kg sorafenib was pretreated through intragastric administration for 2 h before LPS injection. Compared with the LPS group, sorafenib pretreatment restrained the mRNA expression of inflammatory factors IL-6, IL-1β, and TNF-α in lung tissue (Fig. 2A–C), and the ELISA results also showed that soarfenib could decrease the expression of inflammatory factors in mouse serum at 12 h after LPS treatment (Fig. 2D–F). Meanwhile, H&E staining was carried out to observe the injured condition of lung tissue samples (Fig. 2G). Massive inflammatory cells such as macrophages, neutrophils, and lymphocytes infiltrate, perivascular edema, and severe alveolar space destruction were observed following LPS injection compared to the control group, which was proved that the inflammatory mouse model was set up correctly. However, pretreatment with sorafenib significantly reversed the severity of lung injury compared with the mice that received LPS treatment alone. These results indicated that sorafenib inhibits the production of inflammatory factors and alleviates LPS-induced inflammatory response in vivo.

### Sorafenib inhibits phosphorylation of tyrosine kinase Lyn in LPS-treated macrophages

**Fig. 3:**
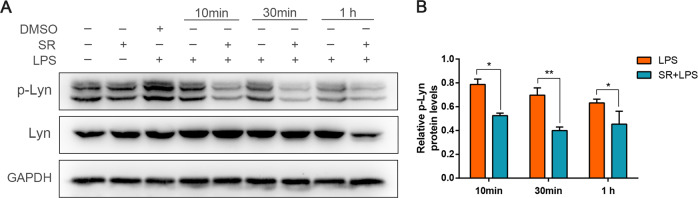
Sorafenib inhibits phosphorylation of tyrosine kinase Lyn in LPS-induced macrophages. BMDMs were pretreated with 10 μmol/L sorafenib for 30 min before incubation with 1 μg/mL LPS for 10 min, 30 min and 1 h. Lyn and the phosphorylation level of Lyn in the whole-cell lysates of BMDMs under the culture conditions as mentioned above were analyzed by western blot analysis. GAPDH used as a loading control **A**. Using gray analysis, the band density quantified in bar graph below as a relative expression compared with loading control **B**. All the images are representative of three independent experiments. The graphs show means ± SEM, *n* = 3; *P < 0.05; **P < 0.01 compared with other groups.

Sorafenib is a novel multiple kinase inhibitor, which targets at the Raf/MEK/ERK signaling pathway and tyrosine kinase receptor related to tumor progression and tumor angiogenesis [21–23]. The phosphorylation of tyrosine kinase plays an important role in regulating TLR4 signals, such as TLR4 tyrosine phosphorylation, p38MAPK, and NF-κB activation in response to LPS [18–20]. However, it is still unclear whether sorafenib can affect LPS-induced inflammatory response through acting on tyrosine kinase Lyn. To study the effect of sorafenib on LPS-induced tyrosine kinase activation, we assessed the level of phosphorylated Lyn (p-Lyn) in the whole-cell lysates by western blot. The results showed that sorafenib significantly inhibited the phosphorylation of Lyn induced by LPS at 10 min, 30 min, and 1 h (Fig. 3A). Interestingly, the time of LYN phosphorylation is short and fades with time, which indicates Lyn acts on the inflammation rapidly. Taken together, the results show that sorafenib inhibits LPS-induced inflammatory reaction, which may be related to the phosphorylation of tyrosine kinase Lyn.

### Sorafenib inhibits phosphorylation of MAPKp38, JNK, and Akt caused by LPS

**Fig. 4:**
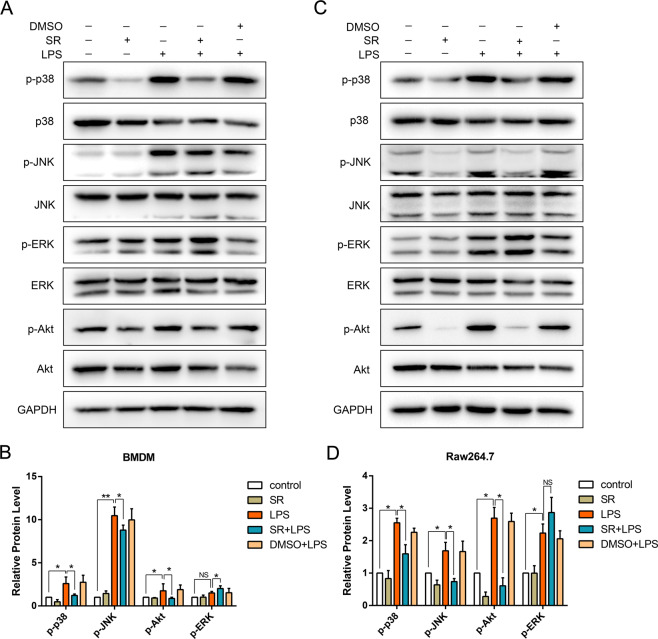
Sorafenib inhibits LPS-induced phosphorylation of MAPKp38 and JNK in macrophage. Immunoblots for p38, phospho (p)-p38, JNK, p-JNK, ERK, p-ERK, Akt, and p-Akt of whole-cell lysates of BMDMs after pretreatment with 10 μmol/L sorafenib followed by LPS (1 μg/mL) stimulation for 1 h **A**. DMSO was used as solvent control group. **C** was verified on Raw264.7 cells in the same way. The band density of each blot was quantified in bar graph as the ratio of phosphorylation (activation) to total protein **B**, **D**. All the images are representative of three independent experiments. The graphs show means ± SEM, *n* = 3; *P < 0.05; **P < 0.01 compared with other groups.

To further investigate the effects of sorafenib on LPS-induced phosphorylation of MAPK pathway protein, the relative protein expression levels of MAPKp38, JNK, and ERK were analyzed by western blot. BMDM and RAW264.7 cells were pretreated with sorafenib at a concentration of 10 μmol/L for 30 min, and then stimulated with 1 μg/mL LPS for 1 h. Levels of both p-p38 and p-JNK increased significantly induced by LPS (Fig. 4A–D), with no increase in protein expression of total p38 or JNK. As expected, sorafenib inhibited phosphorylation of p38 and JNK. At the same time, DMSO of the same volume had no inhibitory effect on p-p38 and p-JNK, which excluded the effect of solvent. Surprisingly, sorafenib did not inhibit the phosphorylation of ERK, but enhanced its phosphorylation level a bit, which may be related to the inhibition of Akt phosphorylation by sorafenib. Collectively, these data indicate that the inhibition of Lyn will affect the activation of MAPK pathway proteins p38 and JNK in its downstream.

### Sorafenib negatively regulates LPS-induced activation of NF-κB/AP-1

**Fig. 5:**
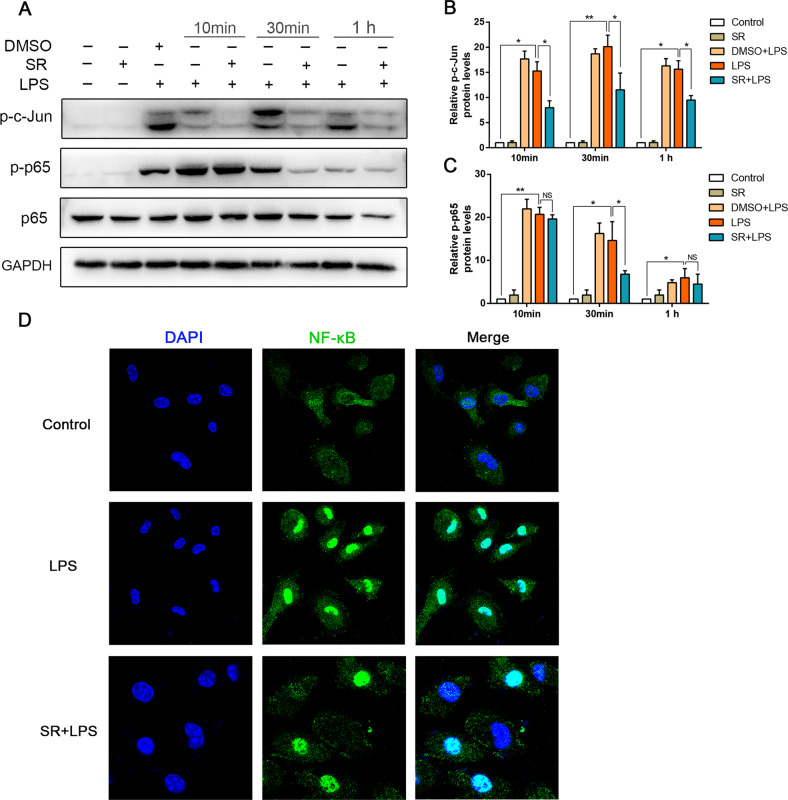
Sorafenib inhibits LPS-induced activation of NF-κB/AP-1. BMDMs were pretreated with 10 μmol/L sorafenib for 30 min before incubation with LPS for 10 min, 30 min, and 1 h. Then the phosphorylation level of NF-κB and AP-1 in the whole-cell lysates of BMDMs were detected by western blot analysis. GAPDH used as a loading control **A**. Using gray analysis, the band density quantified in bar graph below as a relative expression compared with loading control **B**, **C**. Fluorescent microscopy images of NF-κB in BMDMs treated with LPS (1 μg/mL) for 10 min with or without 10 μmol/L sorafenib pretreatment for 30 min **D**. All the images are representative of three independent experiments. The graphs show means ± SEM, *n* = 3; *P < 0.05; **P < 0.01 compared with other groups.

As mentioned above, inflammation response induced by LPS has been inhibited in sorafenib pretreatment. NF-κB and AP-1 signaling pathway is a key to modulate pro-inflammatory cytokine releases. Thus, this classic pathway was also investigated to prove whether NF-κB and AP-1 were involved in our study. As shown in Fig. 5A, LPS increased the phosphorylation level of c-Jun, which was most obvious at 30 min, while sorafenib could significantly inhibit the phosphorylation level of c-Jun stimulated by LPS at each time point. We also found the phosphorylation level of p65 increased most obviously after LPS stimulation for 10 min, decreased after 30 min, and almost completely degraded at 1 h. Sorafenib inhibited phosphorylation of p65 unapparently at 10 min, but most remarkable at 30 min. Using DMSO as the control group, we found that DMSO had no inhibitory effect on the phosphorylation level of c-Jun and p65, which ruled out the interference of solvent. In addition, we also used confocal microscopy to clarify the activation and localization of NF-κB. As shown in Fig. 5D, NF-κB with green fluorescence mainly distributed in the cytoplasm, but rarely distributed in the nucleus in the control group. After LPS stimulation, activated NF-κB with green fluorescence rapidly entered the nucleus, while sorafenib pretreatment decreased distribution of NF-κB in the nucleus. To sum up, sorafenib negative regulation of NF-κB and AP-1 activation serves as one of the major mechanisms that attenuates inflammation in response to LPS.

### Sorafenib downregulates the expression of TLR4 on the membrane in LPS-induced macrophage

**Fig. 6:**
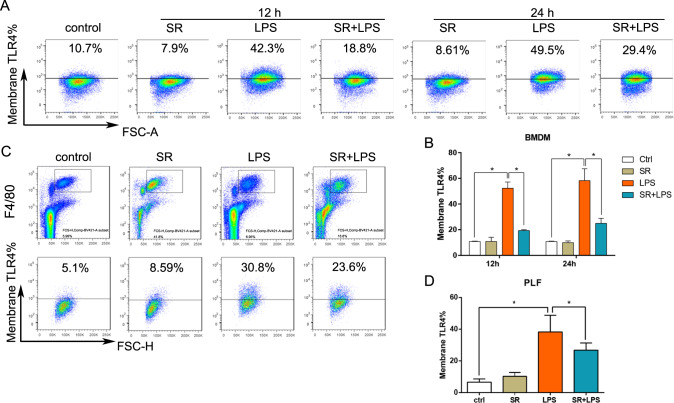
Sorafenib inhibits the expression of TLR4 on the membrane in LPS-induced macrophage. BMDMs isolated from WT mice were treated with LPS (1 μg/mL) in vitro for 6 h, 12 h, and 24 h with or without 10 μmol/L sorafenib pretreatment for 30 min followed by flow cytometry analysis of cell surface TLR4 intensity **A**. TLR4 cell surface expression in peritoneal macrophages (gated by 7AAD—, F4/80 + ) were measured by FACS analysis (**C**). The quantitative analysis bar graph of TLR4 expression on the cell membrane is shown in (**B**, **D**). All images are representative of three independent experiments. The graphs show means ± SEM, *n* = 3; *P < 0.05; **P < 0.01 compared with other groups.

TLR4 is the ligand of LPS, which can be recognized and activated by LPS, and then dimerize the receptor on the cell membrane, initiating the protein-protein cascade reaction, leading to the production of inflammatory cytokines, thus initiating inflammation and immune response [28, 29]. In order to determine whether sorafenib negative regulation of LPS-induced inflammation is related to TLR4, the expression of TLR4 on the membrane were calculated using flow cytometry in vivo and vitro. As shown in Fig. 6A, B, LPS increased the expression of TLR4 on BMDMs membrane compared with the control group, and was positively correlated with stimulation time, while sorafenib pretreatment inhibited its expression. In the same way, 100 mg/kg sorafenib pretreatment by intragastric administration or not, WT (C57BL/6) mice were treated with LPS (20 mg/kg) for time points up to 12 h, after which macrophages were collected from abdominal lavage fluid. The higher expression of TLR4 on the membrane was observed following LPS treatment. Compared with the LPS group, the expression of TLR4 on the membrane of macrophages in the abdominal cavity decreased with sorafenib pretreatment. The results suggested that sorafenib inhibited the expression of TLR4 on the macrophage membrane induced by LPS.

## Discussion

Sorafenib is a multiple kinase inhibitor (MKI) approved for the treatment of primary advanced renal cell carcinoma and advanced primary liver cancer, which is the standard systemic chemotherapy drug for hepatocellular carcinoma (HCC) [30]. Studies have also proved that sorafenib could enhance survival and improve the prognosis in breast cancer, multiple myeloma, and thyroid cancer, etc. [31–33]. Although clinical studies demonstrated some benefits of sorafenib on the time to progression (TTP) and overall survival (OS), its efficacy against HCC remains moderate [34]. The alternative effective treatment regimens or combined treatment is also being explored, which is expected to be a reliable way to treat HCC and ameliorate sorafenib resistance. Uncontrollable inflammation is an important factor to change the microenvironment, which is closely related to the occurrence, development, invasion, and metastasis of tumors. Sorafenib is an effective drug for the treatment of cancer, so what role it plays in the inflammatory mechanism is of great interest to us.

Sorafenib can inhibit tyrosine kinase receptor on the cell surface and serine/threonine kinase in downstream Ras/MAPK cascade signaling pathway of cells. Since these kinases are involved in tumor cell proliferation and tumor angiogenesis, sorafenib is widely used in clinical anti-tumor treatment [35]. Sorafenib is also an effective inhibitor of TNF-dependent necrotic apoptosis through down-regulating the activities of RIPK1 and RIPK3 kinases [36]. Here, we found that sorafenib could significantly inhibit the inflammatory response induced by LPS both in vitro and vivo.

In order to further explore the mechanism of sorafenib-inhibiting LPS-induced inflammatory response, we first observed the tyrosine kinase inhibitory properties of sorafenib. Lyn belongs to a non-receptor tyrosine kinase-cytoplasmic tyrosine kinase. It has been reported that inhibition of tyrosine kinase significantly reduced the production of inflammatory factors such as TNF-α and IL-6 [15, 37, 38]. However, other studies have the opposite conclusion. They found that activation of SFK could inhibit inflammation induced by LPS, or silencing of Lyn upregulated LPS-induced cytokine production [19, 39]. We observed that LPS increased phosphorylation of Lyn, while sorafenib pretreatment could inhibit the activation of Lyn. These results showed that sorafenib inhibited not only the activation of receptor tyrosine kinase, but also the activation of non-receptor tyrosine kinase indicating that sorafenib inhibits LPS-induced inflammatory reaction, which may be partially due to its role in the activation of Lyn.

Mitogen-activated protein kinase (MAPK) transports extracellular signals such as hormones, growth factors, cytokines, and environmental stresses, and triggers various physiological functions, including cell proliferation, differentiation, development, and apoptosis. The three major subfamilies of MAPK include extracellular signal-regulated kinase (ERK), c-Jun N-terminal kinase (JNK), and p38 protein [16, 40, 41]. Previous studies have shown that Lyn knockout affected the activation of MAPK pathway proteins [15]. We proved that sorafenib inhibited the phosphorylation of p38MAPK and JNK protein, which may be caused by the lower phosphorylation level of lyn. However, sorafenib did not inhibit the phosphorylation of ERK, but enhanced its phosphorylation level, which was consistent with the previously reported results [26, 42]. This may be explained as the fact that weakened phosphorylation of Akt promotes active phosphorylation of Raf-1 and subsequent phosphorylation of ERK. As we know, the ubiquitous Raf serine/threonine kinase is the key molecule in the Raf/ MEK/ ERK signaling pathway. Once Raf is activated, MEK will be phosphorylated and activated in Raf subtype, and then phosphorylate and activate ERK [43]. AKT is an inhibitor of Raf-1, which inhibits Raf-1 by phosphorylating Ser259 to create a binding site for the negative regulator of Raf [44]. It, therefore, appeared that sorafenib inhibited the phosphorylation of Akt, decreased the inhibitory effect on Raf, and enhanced the activation of ERK. Further investigation will be required to define the contribution of the different phosphorylation events in p38, JNK, and ERK mediate by sorafenib.

The innate immune response triggered by LPS is mediated by TLR4, which activates transcription factors NF-κB and AP-1 and their nuclear translocation [45]. NF-κB is one of the essential transcription factors in the classical inflammatory pathway, which triggers transcription of inflammatory factors in the recognition of pathogen-related molecular patterns (PAMPs) by pattern-recognition receptors (PRRs) [17, 46]. In an inactive state, NF-κB complex binds to the inhibitory IκB protein in the cytoplasm, which limits its translocation to the nucleus. Activation and phosphorylation of IκB complex promotes the degradation and release of NF-κB, and then the released NF-κB subunits (mainly p65 and p50) are transported to the nucleus to induce the production and release of inflammatory cytokines such as IL-6, IL-1β, and TNF-α [47–49]. AP-1 is one of the other major transcription factors mediated by TLR. Stimulated by LPS, phosphorylation of MAPK proteins lead to the activation and nuclear translocation of AP-1 subunits, such as c-fos, c-jun, ETS domain protein (ELK-2), and activated transcription factor 2 (ATF-2), which combine with DNA reaction elements to promote the release of pro-inflammatory cytokines [50–52]. It was recently proposed that inhibition of src family kinases decreases the phosphorylation of c-Jun, inhibits the activation of IKK-NF-κB signaling pathway, and leads to the formation of AP-1 complex and the decrease of p65 nucleus entry under LPS stimulation, reducing the production and release of inflammatory factors lastly [15, 38, 53]. In addition, phosphorylation of MAPK especially JNK can activate c-Jun [54, 55]. We confirmed that pretreatment of sorafenib inhibited activation of c-Jun and p65 induced by LPS, which may be related to the inhibition of Lyn and MAPK phosphorylation.

The TLR4 signal pathway depends on the cascade reaction of serine–threonine phosphorylation and polyubiquitination, and the activation of TLR4 also triggers off several protein tyrosine kinases, including Syk kinase and SFKs [19, 56]. We discovered that sorafenib can inhibit the expression of TLR4 on macrophages in vitro and in vivo experiments. These data suggest that sorafenib inhibit inflammation response is related to its inhibition of TLR4 expression, and its mechanism may be connected with the inactivation of tyrosine kinase Lyn, which needs to be further studied in the future.

**Fig. 7:**
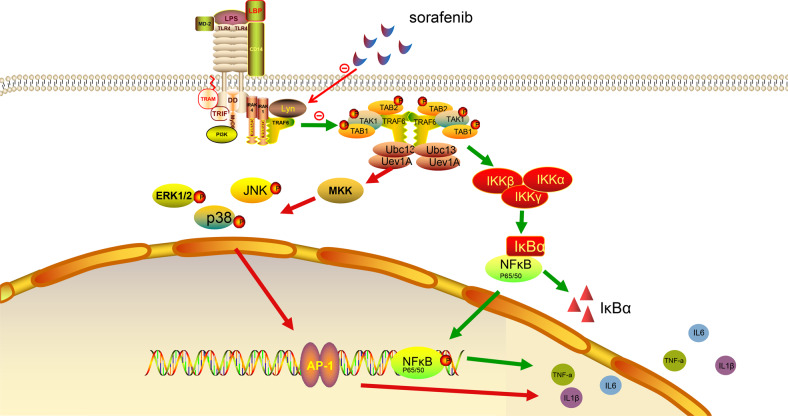
The mechanism of sorafenib-inhibiting inflammation induced by LPS. In the inflammatory reaction induced by LPS, sorafenib inhibits the activation of tyrosine kinase Lyn, and then reduce the phosphorylation of its downstream proteins MAPKp38 and JNK. Sorafenib decreases the activation of c-jun and p65, and then reduces the nucleation of AP-1 and NF-κB, and then reduces the production and release of inflammatory factors such as IL-6, IL-1β, and TNF-α. In addition, sorafenib can also inhibit LPS-induced inflammatory response by inhibiting the expression of TLR4 on the macrophage membrane.

Our major findings in this paper suggest that sorafenib inhibit the phosphorylation of tyrosine kinase Lyn induced by LPS, thereby reducing the phosphorylation level of MAPKp38 and JNK, inhibiting the activation of c-Jun and NF-κB, and then inhibiting the expression of inflammatory factors IL-6, IL-1β, and TNF-α. Furthermore, sorafenib also decreased the expression of TLR4 on the macrophage membrane, which may be related to the inactivation of Lyn. The mechanism diagram is shown in Fig. 7. These results further clarified the effect of sorafenib on LPS-induced inflammation and its mechanism, which provided a new perspective and direction for the clinical treatment of sepsis.

However, there are many limitations in our study. Sorafenib is generally used as an anti-tumor drug in clinic. We have not conducted clinical experiments to explore the efficacy of sorafenib in sepsis patients, whether it is superior to other antibiotics, and so on. These are the topics that we are going to carry out in the later stage. In terms of mechanism, it is necessary to further clarify the specific mechanism of sorafenib inhibiting the expression of TLR4 on the membrane, whether it is related to inhibiting the transport of TLR4 from endoplasmic reticulum to cell membrane or decreasing the transcription and translation of TLR4. In addition, it is interesting that sorafenib enhances the phosphorylation of ERK induced by LPS and it may also be the direction of further exploration in the future. Furthermore, inflammation is closely related to oxidative stress, and sorafenib has been proved to be a ferroptosis inducer, so how the effect of ferroptosis induced by sorafenib is related to its inhibition of LPS-induced inflammatory response will be a new field worthy of further study.

## Materials and methods

### Cells culture and treatment

BMDM isolation and culture refer to the methods of previous study [57]. Bone marrow from male mice (6–8 weeks) was flushed out and cultured in DMEM containing 10% FBS complemented with 50 mg/ml penicillin/streptomycin and 10 ng/ml M-CSF (PeproTech, Lot#0817245). After 5–7 days of culture, BMDM differentiated and matured, and then changed into fresh medium (DMEM containing 10% FBS) for dosing treatment. Subcultured Raw264.7 murine macrophage was obtained from the ATCC (Bethesda, MD, USA). Cells were also cultured in DMEM containing 10% FBS(Gibco, USA), streptomycin (100 μg/mL, Life Technologies), and penicillin (100 units/ml, Life Technologies) and incubated overnight at 37 °C and 5% CO_2_. Sorafenib was prepared in dimethyl sulfoxide (DMSO) and diluted to the desired final concentration (0, 5, 10 μmol/L) in a culture medium for 30 min before LPS (1 μg/mL) treatment. The final concentration of DMSO did not exceed 0.1%.

### Animals

Fifty C57BL/6 male (wild-type; WT) mice (6–8 wk old), weight range 20–22 g, were purchased from Beijing Huafukang Biotechnology (License No. SCXK (Jing) 2019). All the animal experimental protocols were reviewed and conformed to the committees of Guangdong Medical University. For animal studies, the mice were divided into the following four groups: (1) control group: mice received intraperitoneal (i.p.) injections of saline for 12 h; (2) sorafenib group: 100 mg/kg sorafenib(dissolved in 0.5% CMC-Na) for intragastric administration 2 h before saline i.p. 12 h; (3) LPS group: mice were treated with LPS (20 mg/kg, i.p.) for 12 h; (4) LPS + sorafenib group: mice were pretreated with 100 mg/kg sorafenib orally 2 h before LPS (20 mg/kg, i.p.) treatment 12 h.

### Antibodies and reagents

Lipopolysaccharides (LPS) were purchased from Solarbio (Lot:L8880). Sorafenib (BAY 43-9006, CAS No.284461-73-0), and CMC-Na (Sodium carboxymethyl cellulose, CAS No. 9004-32-4) were purchased from Selleck. Abs specific for Phospho-Lyn (70926S), Lyn (4576S), Phospho-p38 (4631S), p38 (8690S), Phospho-Erk1/2 (9101S), Erk1/2 (9102S), Phospho-JNK (9255S), JNK (9252S), Phospho-Akt (4060S), Akt (4691S), Phospho-p65 (3033S), p65 (8642S), and Phospho-c-Jun (4691S) were obtained from Cell Signaling Technology. GAPDH (Cat No. 60004-1-Ig) was purchased from Proteintech.

### Flow cytometry analysis

Macrophages collected from peritoneal lavage were labeled with F4/80 at 4 °C for 15 min. For measuring cell surface expression of TLR4, BMDMs and/or PLF cells were stained with PE-conjugated anti-mouse TLR4-PE (12-9041-80, 1:200; eBioscience) for 30 min. Cells were washed three times with PBS, after suspension in PBS, and then cells were analyzed by flow cytometry (BD Biosciences). Data were analyzed using FlowJo software (Tree Star).

### RNA extraction and quantitative real-time PCR

Total cellular RNA was extracted using TRIzol reagent (Invitrogen). Real-time RT-PCR was done using the iTaq™ Universal SYBR^®^ Green Supermix (172-5121, Bio-Rad, Hercules, CA, USA) in a Bio-Rad iQ5 real-time PCR machine (Bio-Rad). Reverse transcription was done using iScript Reverse Transcription Supermix (170-8841, Bio-Rad, Hercules, CA, USA). Amplification was performed with cycling conditions of 95 °C for 15 s then 60 °C for 30 s for 40 cycles. After the amplification protocol was over, the PCR product was subjected to melt-curve analysis using Bio-Rad iQ5 software (Bio-Rad). The real-time PCR primers were synthesized as follows:

β-actin (forward: CATGTACGTTGCTATCCAGGC;

reverse: CTCCTTAATGTCACGCACGAT).

IL-6 (forward: TCTATACCACTTCACAAGTCGGA;

reverse: GAATTGCCATTGCACAACTCTTT).

TNF-α (forward: AAGCCTGTAGCC CACGTCGTA;

reverse: GGCACCACTAGTTGGTTGTCTTTG);

IL-1β (forward: GCAACTGTTCCTGAACTCAACT;

reverse: ATCTTTTGGGGTCCGTCAACT).

### Western blotting

Total proteins were extracted from the BMDM or Raw264.7 cells with the lysis buffer supplemented with 1 mM PMSF, and a protease inhibitor cocktail. Protein concentration determined by the bicinchoninic acid (BCA) protein assay (Beyotime). Proteins were subjected to SDS/PAGE, transferred onto polyvinylidene difluoride (PVDF) membranes, and blotted with 5% skimmed milk. The membranes were incubated overnight with primary antibodies against GAPDH, Phospho-Lyn, Total-Lyn, Phospho-p38, Total p38, Phospho-Erk1/2, Total-Erk1/2, Phospho-JNK, Total-JNK, Phospho-Akt, Total-Akt, Phospho-p65, p65, and Phospho-c-Jun, respectively. Horseradish peroxidase-conjugated anti-rabbit IgG was used as the secondary antibody.

### Enzyme-linked immunosorbent assay (ELISA)

IL-6, IL-1β, and TNF-α concentrations in mouse serum were measured using murine cytokine-specific Quantikine ELISA kits (eBioscience) according to the manufacturer’s instructions.

### Statistical analysis

Data were analyzed in SPSS19.0 (Carlsbad, CA). For multi-group comparisons, One-way ANOVA was performed. All graphs represent mean ± s.e.m. *P* < 0.05 was considered statistically significant. Graphs and figures were made with GraphPad Prism 5 (GraphPad software, Carlsbad, CA).

## Supplementary information

western blot

## Data Availability

The data that support the findings of this study are available from the corresponding author on reasonable request.
