# Supplementary material for: Sorafenib inhibits LPS-induced inflammation by regulating Lyn-MAPK-NF-kB/AP-1 pathway and TLR4 expression
Source: Cell Death Discov. 2022 Jun 9;8:281. doi: 10.1038/s41420-022-01073-7 (PMC9184561; doi:10.1038/s41420-022-01073-7)

Figure3

Lyn

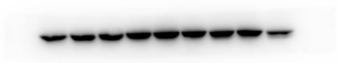

gapdh

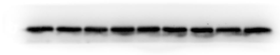

p-Lyn

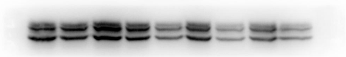

Lyn

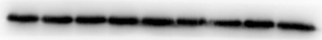

p-Lyn

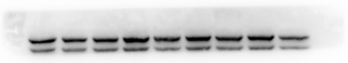

Figure4

BMDM

p-Lyn

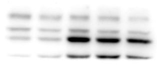

p-AKT

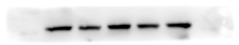

JNK

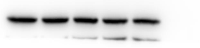

ERK

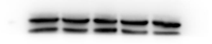

p-erk

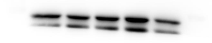

P38

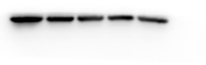

gapdh

AKT

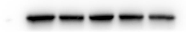

p-p38

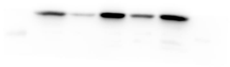

RAW264.7

p38

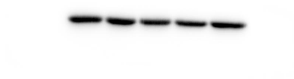

p-p38

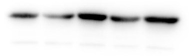

JNK

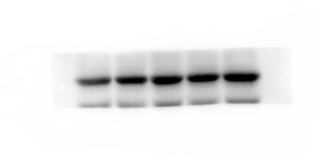

GAPDH

ERK

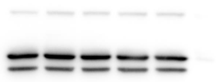

AKT

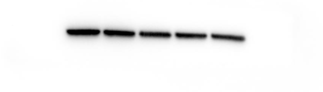

p-JNK

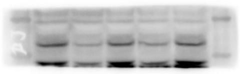

p-ERK

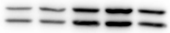

p-AKT

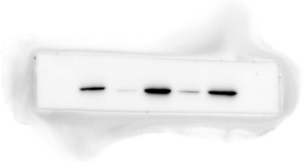

Figure5

p-c-jun

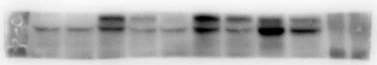

P65

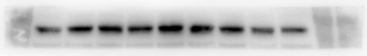

gapdh

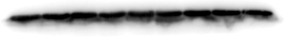

p-p65

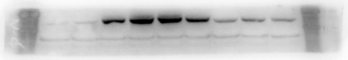

p-c-Jun

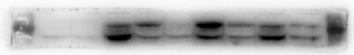

P65

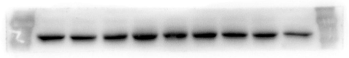

gapdh

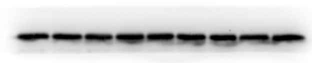

p-p65

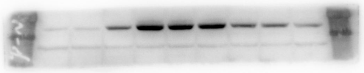

Supplement: Supplementary file 1 — western blot [file 41420_2022_1073_MOESM1_ESM.pdf]
